# Distinct mechanisms of loss of IFN-gamma mediated HLA class I inducibility in two melanoma cell lines

**DOI:** 10.1186/1471-2407-7-34

**Published:** 2007-02-23

**Authors:** Teresa Rodríguez, Rosa Méndez, Ana Del Campo, Pilar Jiménez, Natalia Aptsiauri, Federico Garrido, Francisco Ruiz-Cabello

**Affiliations:** 1 Servicio de Análisis Clínicos, Hospital Universitario Virgen de las Nieves, Granada, Spain

## Abstract

**Background:**

The inability of cancer cells to present antigen on the cell surface via MHC class I molecules is one of the mechanisms by which tumor cells evade anti-tumor immunity. Alterations of Jak-STAT components of interferon (IFN)-mediated signaling can contribute to the mechanism of cell resistance to IFN, leading to lack of MHC class I inducibility. Hence, the identification of IFN-γ-resistant tumors may have prognostic and/or therapeutic relevance. In the present study, we investigated a mechanism of MHC class I inducibility in response to IFN-γ treatment in human melanoma cell lines.

**Methods:**

Basal and IFN-induced expression of HLA class I antigens was analyzed by means of indirect immunofluorescence flow cytometry, Western Blot, RT-PCR, and quantitative real-time RT-PCR (TaqMan^® ^Gene Expression Assays). In demethylation studies cells were cultured with 5-aza-2'-deoxycytidine. Electrophoretic Mobility Shift Assay (EMSA) was used to assay whether IRF-1 promoter binding activity is induced in IFN-γ-treated cells.

**Results:**

Altered IFN-γ mediated HLA-class I induction was observed in two melanoma cells lines (ESTDAB-004 and ESTDAB-159) out of 57 studied, while treatment of these two cell lines with IFN-α led to normal induction of HLA class I antigen expression. Examination of STAT-1 in ESTDAB-004 after IFN-γ treatment demonstrated that the STAT-1 protein was expressed but not phosphorylated. Interestingly, IFN-α treatment induced normal STAT-1 phosphorylation and HLA class I expression. In contrast, the absence of response to IFN-γ in ESTDAB-159 was found to be associated with alterations in downstream components of the IFN-γ signaling pathway.

**Conclusion:**

We observed two distinct mechanisms of loss of IFN-γ inducibility of HLA class I antigens in two melanoma cell lines. Our findings suggest that loss of HLA class I induction in ESTDAB-004 cells results from a defect in the earliest steps of the IFN-γ signaling pathway due to absence of STAT-1 tyrosine-phosphorylation, while absence of IFN-γ-mediated HLA class I expression in ESTDAB-159 cells is due to epigenetic blocking of IFN-regulatory factor 1 (IRF-1) transactivation.

## Background

Interferon gamma (IFN-γ) is a pro-inflammatory pleiotropic cytokine that plays a central role in promoting innate and adaptive mechanisms of host defense [1,2]. Interferon IFN-γ secreted by T and natural killer (NK) cells is important in driving T helper cell type 1 (Th1) responses. In addition, IFN-γ plays a key role in providing an immunocompetent host with a mechanism of tumor surveillance [3]. Specific antitumor immune responses require expression of MHC class I on tumor cells, and MHC antigen down-regulation is a presumed tumor growth promoting mechanism [4]. However, there is experimental evidence of tumors showing dysregulation of multiple signaling pathways that interfere with cytokine signals [5,6]. Certain tumors may lose IFN-γ sensitivity as a mechanism to evade immune detection [7]. IFNs exert their effects by binding cell surface receptors, triggering an intracellular signaling cascade of Janus kinases (Jak) and signal transducers and activators of transcription (STAT) proteins, which results in the transcription of IFN-inducible genes. IFN-γ exerts its effects after binding to a receptor composed of two heterodimeric receptor subunits, IFN-γ R1 and IFN-γ R2, which are intracellularly associated with Janus kinases (Jaks) Jak-1 and Jak-2, respectively [8,1]. The binding initiates receptor oligomerization and phosphorylation of tyrosine residues in Jak1, Jak2, and the cytoplasmic tail of IFN-γ R1. Each phosphorylated IFN-γ R1 chain becomes a docking site for STAT-1. After docking at the receptor, STAT-1 phosphorylated on tyrosine 701 undergoes dimerization and translocates to the nucleus, where it binds the IFN-γ activation sequence (GAS) elements present in the promoters of IFN-γ-inducible genes. IFN-γ is one of the strongest inducers of IRF-1 via activation of STAT-1 and binding to the GAS sequence within the IRF-1 promoter. In addition, IFN-γ upregulated HLA class I expression via a STAT-1/IRF1-dependent pathway. HLA class I promoter contains the IFN-γ-responsive element (IRSE) [9], which is a binding site for factors of the IRF family and mediates the induction of MHC class I transcription by IFNs. IFN-γ has an additional rout, distinct from that of IFN-α, for upregulation of HLA class I via class II transactivator (CIITA) that binds the α-site of HLA class I promoter [9]. Type-I-IFN induced Jak-STAT signaling is propagated similarly to IFN-g-induced Jak-STAT signaling. Activated TYK2 and JAK1 phosphorylate STAT1 and STAT2. Type-I-IFN-mediated signaling then induces homodimerization of STAT1 and heterodimerization of STAT1 and STAT2 (in case of IFN-γ only dimerization of Stat1 takes place) which subsequently associate with the cytosolic transcription factor IFN-regulatory factor 9 (IRF9), forming a trimeric complex known as IFN-stimulated gene factor 3 (ISGF3). On entering the nucleus, ISGF3 binds IFN-stimulate response elements (ISREs) [10]. IFN-α-mediated MHC class I induction involves two protein families, the STATs and IRFs, which form the interferon-stimulated transcription factor-3 (ISGF3) complex that also binds the IRSE sequence.

Because immunotherapy is an important approach to melanoma treatment, it is important to understand the mechanisms by which these tumors circumvent IFN signaling, thereby representing a potential mechanism of melanoma cytokine-resistance to therapy. The aim of this study was to evaluate the expression of HLA class I antigens in response to IFN-γ in 57 human melanoma cell lines. Defects in the inducibility of HLA class I antigens in response to IFN-γ and normal response to IFN-α induction were observed in two of the tested melanoma cell lines (ESTDAB-004 and ESTDAB-159).

## Methods

### Cell culture and reagents

Fifty seven melanoma cell lines were obtained from the European Searchable Tumor Cell Line Data Base (ESTDAB project, contract no.QLRI-CT-2001-01325) [11]. Melanoma cell lines were grown in RPMI medium (Biochrom KG, Berlin, Germany) supplemented with 10% fetal calf serum (FCS; Gibco BRL, Life Technologies, Karlsruhe, Germany), 2% glutamine (Biochrom KG, Berlin, Germany), and 1% penicillin/streptomycin (Biochrom KG, Berlin, Germany) at 37°C in a humidified atmosphere with 5% CO_2_. Cell lines were treated with IFN-γ or IFN-α for 48 h. Recombinant IFN-γ and IFN-α were purchased from R&D Systems (Minneapolis, USA). For demethylation studies, cells were resuspended in fresh complete medium (5 × 10^6^cells/ml) and cultured with 3 μM of 5-aza-2'-deoxycytidine (Sigma-Aldrich, Madrid, Spain) for 7 days.

Responsiveness of human tumor cells to IFN was assessed after incubation of the cells in the presence of IFN-γ (800 IU/ml) or IFN-α (800 IU/ml) for 48 h followed by FACS analysis of MHC class I antigen expression. PBS was used as a control. Anti-IRF-1, and consensus IRF-1 gel shift oligonucleotides were obtained from Santa Cruz Biotechnology, Inc. (Santa Cruz, CA, USA). Monoclonal antibodies against IRF-1 and STAT-1 were obtained from Transduction Laboratories (Lexington, KY, USA). Polyclonal antibody against phospho- STAT-1 at residue Tyr-701 was obtained from Cell Signaling Technology (Beverly, MA, USA). Rabbit anti-SOCS-1 polyclonal antibody (AnaSpec, Inc, San Jose, CA, USA) and an anti-Phospho-Jak2 (Tyr1007/1008) antibody (Cell Signaling Technology, USA) were also used.

### HLA cell surface expression

Surface expression of HLA class I antigens was determined by indirect immunofluorescence using a panel of monoclonal antibodies, W6/32 (HLA-ABC), L-362 (β2 m), 1082C5 (HLA-A), Q6/64 (HLA-B), 126.39 (HLA-Bw6), and 116-5-28 (HLA-Bw4). 10^5 ^cells were treated with IFN at 800 IU/ml for 48 h. Cells were harvested using buffered EDTA solution, then washed with RPMI medium containing 10% v/v FCS and 0.1% w/v sodium azide (medium/FCS/azide) prior to incubation with primary antibody (diluted in medium/FCS/azide) for 60 min at 4°C in the dark. After incubation with unlabeled primary antibody, the cells were washed, incubated with FITC-labeled secondary antibody for 45 min at 4°C, and then washed again. Flow cytometry was performed using BD FACSort (Becton Dickinson, Oxford, UK). Ten thousand events were captured and analyzed using CellQuest software (Becton Dickinson).

### Western Blot Analysis

The melanoma cell lines were treated with IFN-α and IFN-γ for different periods of time and cultured in medium containing 0.5% FCS for 2 h before lysing with 1X SDS sample buffer (62.5 mM Tris-HCl, 2% w/v SDS, 10% glycerol, 50 mM DTT, 0.01% w/v bromophenol blue). Proteins from the whole cell lysates were separated by polyacrylamide gel electrophoresis and transferred to nitrocellulose membranes, which were then sequentially incubated in primary antibody overnight at 4°C and then with HRP-conjugated secondary antibody (1:2000) in blocking buffer for 1 h at room temperature. Protein extracts from whole-cell lysates were probed for monoclonal antibodies that recognize STAT1 or phospho-Y701 STAT1 (Cell Signaling, Beverly, MA, USA). Before incubating with primary antibodies, membranes were placed in a blocking buffer containing 1X TBS, 0.1% Tween-20 and 5% nonfat dry milk for 1 h at room temperature. The dilution buffer for the primary and secondary antibodies contained 1XTBS, 0.1 Tween-20 and 5% BSA. For detection of immunolabeled proteins, membranes were incubated with 0.5 ml 20X LumiGLO and 0.5 ml 20X peroxide in 9 ml of water with gentle agitation for 1 min at room temperature and then transferred to X-ray film. Anti-γ-tubulin antibody was used to normalize the protein content in each well of gel electrophoresis. As a positive control we used the extract from cell line A549 that came as a part of the anti-phospho-STAT1 antibody kit (Cell Signaling, Beverly, MA, USA).

### RNA isolation and RT-PCR

Total cellular RNA was isolated using a Qiagen RNeasy kit (Qiagen GmbH, Hilden, Germany) according to the manufacturer's instructions. Total RNA (0.5 μg) was subjected to reverse transcription, and one-tenth of the reaction mixture was used for PCR analysis. First strand cDNA was synthesized with 10 μl of total RNA, using Sensiscript reverse transcriptase kit (Quiagen) with 40 U/μl of RNasin (Promega, Madison, WI, USA) and random primers (Promega Madison, WI, USA) in a final volume of 20 μl. The reaction was incubated at 42°C for 60 min and stopped at 95°C for 5 min. Time-course effect of IFN-γ on the mRNA expression of IRF-1, iNOS, and HLA-B in ESTDAB-159 were measured. The following primer pairs were used: 5- CGG CCT TAA GAA CCA GGC AAC CT-3 (forward) and 5-CCA GCT TCT CTG CAC CAT ATC CA-3 (reverse), for differential expression of IRF-1 mRNAs; 5- GAG CTT CTA CCT CAA GCT ATC-3 (forward) and 5-CCT GAT GTT GCC ATT GTT GGT -3 (reverse) for iNOS mRNA: and 5-GAC AGC GAC GCC GCG AGT CC -3 (forward) and 5- AGT AGC GAC CAC AGC TCC GA-3 (reverse) for HLA-B mRNA. Glyceraldehyde-3-phosphate dehydrogenase (GAPDH) was used as an internal control. The primers for GAPDH were 5 -TGA AGG TCG GAG TCA ACG GAT TTG GT-3 (forward) and 5 -CAT GTG GGC CAT GAG GTC CAC CAC-3 (reverse).

### Real-Time PCR

RT-PCR products were analyzed by quantitative real-time RT-PCR in TaqMan^® ^Gene Expression Assays of several target genes: HLA-A, HLA-B heavy chain, HLA-A (Hs 00740413-g1), HLA-B (Hs 00818803-g1), and beta glucuronidase (GUSB; as an endogenous control) (Applied Biosystems; Foster City, CA, USA) for variations in the amounts of RNA. All PCR reactions were performed in a real time PCR 7500 system. Gene expression quantitation using TaqMan^® ^Gene Expression Assays was performed as the second step in a two-step RT-PCR. Assays were done in 20-μL singleplex reactions containing TaqMan^® ^Universal PCR Master Mix, 20X TaqMan^® ^Gene Expression Assay Mix, and cDNA according the manufacturer's instructions (Applied Biosystems). Reaction conditions consisted of pre-incubation at 50°C for 2 min, 95°C for 10 min, then cycling for 40 cycles of 95°C for 15 sec and 60°C for 1 min.

### Electrophoretic Mobility Shift Assay

Nuclear extract was prepared as previously described [12,13] in the presence of protease inhibitors. Extracts were aliquoted and stored at -80°C. For each electrophoretic mobility shift assay reaction, 4 μg of nuclear proteins were incubated with 30,000 cpm of [γ-^32^P]ATP-labeled consensus IRF-1 gel shift oligonucleotide (Santa Cruz Biotechnology, Inc., Santa Cruz, CA) for 30 min on ice. In competition assay unlabeled IRF-1 consensus or anti-IRF-1 monoclonal antibody (2 μg) of rabbit polyclonal antisera against IRF-1 (Santa-Cruz Biotechnology, USA) were incubated with nuclear extracts for 10 min on ice prior to the addition of radiolabeled oligonucleotides. Reaction mixtures were then separated on a 5% native polyacrylamide gel. After electrophoresis, the gel was dried and subjected to autoradiography.

## Results

### Loss of IFN-γ-mediated MHC class I inducibility in melanoma cell lines

**Figure 1:**
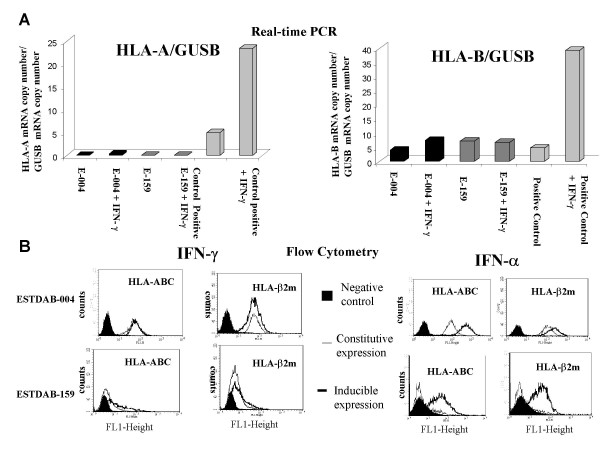
Transcription level (A) and cell surface expression (B) of HLA-ABC molecules in ESTDAB-004 and ESTDAB-159 melanoma cell lines [in (B) examined by indirect immunofluorescence using W6/32 and L-362 mAb]. Cells were treated with 800 U/ml of either IFN-γ or IFN-α for 48 h or with culture medium alone. The dark histogram corresponds to the isotypic control. Panel **A **– the results of real-time RT-PCR analysis. On the Y-axis on panel **A **– mRNA copy number of the gene of interest (HLA-A or HLA-B) normalized against the mRNA copy number of the reference gene GUSB.

Cell surface expression of HLA class I molecules was analysed in a panel of 57 melanoma cell lines by flow cytometry. The response of MHC class I expression to IFN-treatment varied. Almost all of the melanoma cell lines showed increased surface expression of HLA class I antigens after 48 h of culture. Only four melanoma cell lines did not increase MHC class I expression after incubation with IFN-γ. Since two of them had β2 microglobulin mutation [14], they were not included in this study. However, the other two lines (ESTDAB-004 and ESTDAB-159) (Fig. 1) with absence of IFN-γ mediated increase in expression of the HLA class I molecules demonstrated HLA class I induction after treatment with IFN-α. Loss of IFN-γ mediated inducibility was also observed in MHC class II molecules (not shown). MHC class I expression in these two cell lines was also analysed by quantitative RT-PCR, and no upregulation of MHC class I mRNA was detected (Fig 1A). Treatment of both cell lines with 800 U/ml IFN-γ for 48 h produced no significant increase in the expression of MHC class I mRNA in comparison with a control cell line (ESTDAB-052, which responded with HLA-class I induction) (Fig. 1A). This finding was consistent with the protein expression as demonstrated by FACS analysis (Fig. 1B). Loss of IFN-γ mediated class I inducibility was not caused by a post-transcriptional defect. Because the IFN-γ signal transduction pathway shares two intracellular signalling molecules with the IFN-α pathway, these cells were also tested for their ability to up-regulate MHC class I in response to IFN-α. Both melanoma cell lines responded to IFN-α treatment with an increased MHC class I cell surface expression, indicating the possibility of a specific and selective alteration only in the IFN-γ signalling pathway.

### IFN-γ resistant cell lines (ESTDAB-004 and ESTDAB-159) have different patterns of alteration in the IFN-γ signaling pathway

**Figure 2:**
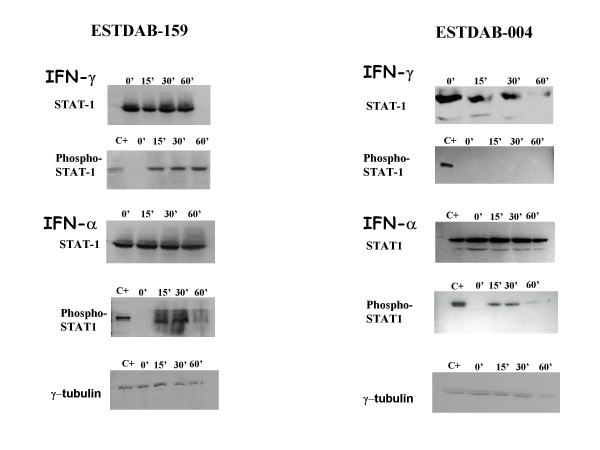
The expression and the activation of STAT proteins. Cells were treated with IFN-γ or IFN-α at 800 IU/ml. Proteins from cellular protein extracts were separated by SDS-PAGE and transferred to nitrocellulose membrane. Western blotting was performed as described in Materials and Methods. Anti-STAT1 and anti-phospho-STAT1 antibodies were used to assess STAT activation. Anti-γ-tubulin antibody was used to normalize the amounts of protein loaded in each well of the gel. Positive control (C+) of phosphorylated proteins from cellular extract A549 was obtained from Cell Signaling Technology.

**Figure 3:**
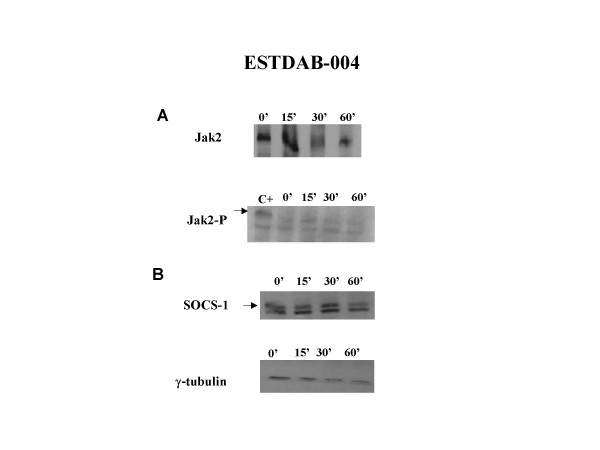
Western blot analysis of the expression level and phosphorylation status of Jak-2 (A), and of the expression of SOCS-1 (B) in ESTDAB-004 cell line. We observed that Jak-2 was present but its phosphorylation was very low and was accompanied by a high SOCS-1 expression (negative regulator of IFN-γ response). Arrows indicate the specific proteins. Nuclear extracts as a positive control (C+) for Jak-2 were obtained from Cell Signaling.

STAT-1 phosphorylation was examined by western blot using anti-STAT-1 or anti -phospho-STAT-1 antibodies that recognize unphosphorylated or phosphorylated STAT-1, respectively. Cells were incubated with 800 U/ml IFN-γ or IFN-α before western blot analysis. STAT-1 induction and activation were analyzed in both cell lines to yield further information on the possible mechanism of altered response to IFN-γ treatment. As shown in Figure 2, STAT-1 tyrosine phosphorylation occurred only in the ESTDAB-159 cells, whereas phosphorylation was undetectable in ESTDAB-004 cells. STAT-1 phosphorylation was upregulated and rapidly decreased after 60 min of incubation with IFN-γ. However, both cell lines responded with STAT-1 phosphorylation to IFN-α treatment (Figure 2). The tyrosine phosphorylation of Jak-2 in response to IFN-γ stimulation was also investigated. Absence of phosphorylation in response to IFN-γ was observed in ESTDAB-004 cells, which was correlated with decreased IFN-γ signaling (Figure 3). Suppressor of cytokine signaling-1 (SOCS1) is a critical negative regulator of IFN-γ responses. Its expression is induced in response to stimulation of a variety of cytokines, and over-expression of this protein results in inhibition of cytokine signaling [15]. SOCS1 protein expression was detected in ESTDAB-004 melanoma cells without treatment with IFN-γ and did not change after this treatment (Figure 3).

### IRF-1 is upregulated in ESTDAB-159 cells in response to IFNγ

**Figure 4:**
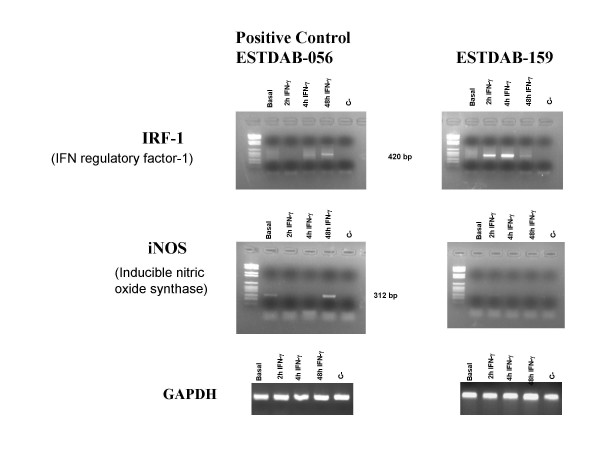
Time course effect of IFN-γ on the expression of IRF-1 and iNOS (A). Total RNAs were isolated from ESTDAB-159 cell line and form the control cell line ESTDAB-056 grown in the presence or absence of 800 U/ml IFN-γ for the indicated periods of time and analyzed for the relative levels of mRNA by RT-PCR.

After tyrosine-phosphorylation, STATs form homo- or heterodimers and translocate from the cytoplasm to the nucleus, where they bind specific DNA sequences and activate transcription of many genes. IRF-1 is transactivated by binding of STAT-1 dimers to the GAS sequence of the IRF-1 promoter. IRF-1 is the principal factor mediating the IFN-γ-induced expression of MHC class I antigen. In order to analyze the Jak-STAT signalling cascade, we investigated the induction of IRF-1 mRNA expression in response to IFN-γ treatment. As shown in Figure 4A, IRF-1 mRNA was upregulated by IFN-γ treatment in ESTDAB-159 cells. We also investigated the expression of another IRF-1- regulated gene, iNOS, to determine whether MHC class I gene expression is selectively blocked. We measured iNOS mRNA level in IFN-γ-treated ESTDAB-159 cells. As shown in Fig 4B, IFN-γ did not induce expression of iNOS mRNA, indicating an inhibition of IRF-1 transactivated genes.

### IRF-1 binding activity is induced in INF-γ-treated ESTDAB-159 cells

**Figure 5:**
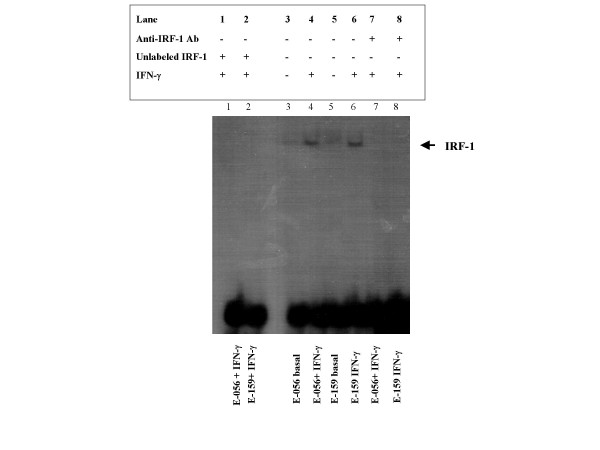
EMSA showing the binding of IRF-1 to a ^32^P labeled probe containing the IRF-1 consensus sequence. EMSA was performed with nuclear extracts obtained from untreated ESTDAB-056 (3) and ESTDAB-159 (5) melanoma cells or treated with 800 U/ml IFN-γ for 4 h (lanes 1, 4 and 7 corresponding to ESTDAB-056; and lanes 2, 6 and 8 corresponding to ESTDAB-159). A 50-fold molar excess of unlabeled IRF-1 probe was added to the binding reaction (lane 1, 2) to compete out the formation of a detectable complex. Anti-IRF-1 antibody was used to block IRF-1 binding to test the specificity of the interaction (lanes 7 and 8). Results shown are representative of at least three independent experiments.

IFN-γ-stimulated IRF-1 induction was assayed using electrophoretic mobility shift assay (EMSA). As shown in Fig. 5, IRF-1 binding to the consensus element was induced after IFN-γ stimulation of ESTDAB-159 and of control cell line ESTDAB-056. Protein complex formation between IRF-1 and DNA probes was increased in the nuclear extract of IFN-γ-treated melanoma cells (lanes 3, 4, 5 and 6). Formation of detectable complex was competed out with 50-fold excess of unlabeled cold probe (lanes 1, 2). Supershift experiments using an anti-IRF-1 antibody (lanes 7, 8) indicated that the complex was specific. These results strongly suggest that the nuclear protein complex that binds to the putative IRF-1 binding probe contains a functional IRF-1 protein.

### 5-Aza-2-deoxycytidine restores IFN-γ-mediated HLA-class I inducibility in ESTDAB-159 cells

**Figure 6:**
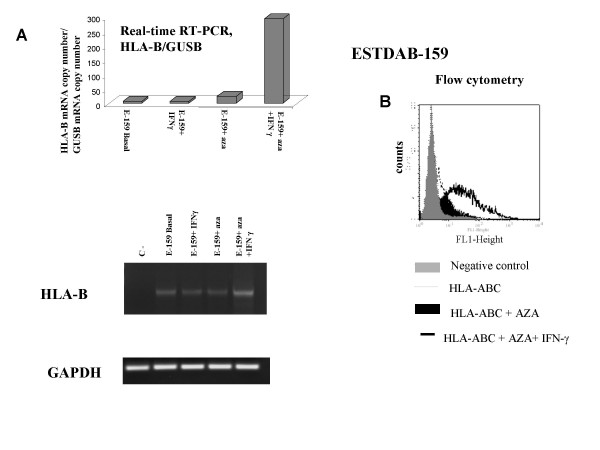
5-Aza-2-deoxycytidine (5-dAzaC) recovers IFN-γ mediated HLA-class I inducibility in ESTDAB-159 melanoma cells. Cells were untreated or pretreated for 7 days with 3 μM 5-dAzaC and/then untreated or incubated with 800 U/ml IFN-γ for 48 hrs. HLA class I expression before and after treatment with 5-dAzaC was determined by flow cytometry analyzes of HLA-ABC surface expression using W6/32 monoclonal antibody (B), and by quantitative RT-PCR (A). Results of HLA-B expression are normalized against GUSB expression (HLAB/GUSB).

We demonstrated the role of methylation in the loss of the inducibility of HLA class I expression by incubating ESTDAB-159 cells with the demethylating substance 5-Aza-2'-deoxycytidine. After 7 days of culture, 5-dAzaC restored IFN-γ-induced HLA-class I expression. Treatment of cells with either 5-dAzaC or IFN-γ alone did not lead to increase (or only to a marginal increase) in HLA class I protein or mRNA expression, respectively (Figure 6A). Interestingly, administration of both 5-dAzaC and IFN-γ produced an increase in the level of transcripts and led to cell surface expression of HLA class I molecules, as revealed by quantitative RT-PCR and flow cytometry analysis (Figure 6B).

## Discussion

IFNs were the first cytokines to be used in clinical trials for cancer treatment. IFNs have significant effects and play a central role in providing an immunocompetent host with a mechanism of tumor surveillance and in promoting innate and adaptive host defense [3]. Moreover, IFN-γ may have an anti-metabolic and inhibitory effect on tumor cell growth in vitro, as has been shown in various cell types, including malignant melanoma [16,17]. Importantly, for tumor rejection to occur, antigens must be presented in a complex with HLA class I molecules on the melanoma cell surface to be recognized by CTLs. In this context, melanoma cells may attempt to escape cell-mediated immunosurveillance in order to develop a permanent and selective IFN-γ insensitivity by inhibiting expression of MHC molecules.

Many studies highlight the importance of Jak-STAT pathways in IFN resistance. Interestingly, melanomas differ in their responsiveness to IFNs and can be IFN-sensitive or IFN-resistant. IFN-resistant melanoma cells contain reduced levels of ISGF3a components, particularly of STAT-1, indicating that the non-responsiveness of melanoma cell lines to type I IFN may be due to a deficiency in the expression of ISGF3 components [7]. An absence of response to IFN in lung and prostate adenocarcinoma cell lines has been attributed to lack of Jak-1 gene expression [18]. We also previously reported a deficiency in STAT-1 component as a probable cause of resistance to IFNs detected in a gastric carcinoma cell line [12,13]. In the present report, our results suggest heterogeneity in the mechanisms underlying IFN resistance in melanomas. We analyzed MHC class I antigen inducibility after IFN-γ treatment in 57 human melanoma cell lines. Only four cell lines showed no responsiveness to IFN by flow cytometry, and two of these were excluded from the study because they presented a mutation in β2 m gene [19,14] and recovered IFN-γ inducibility after reconstitution of wild type β2 m gene (not shown). The remaining two cell lines (ESTDAB-004 and ESTDAB-159) exhibited specific resistance to IFN-γ but responded normally to IFN-α treatment; the mechanism underlying IFN-γ resistance differed between these two cell lines.

In ESTDAB-004 cells, our findings strongly suggest that defective activation of STAT-1 was responsible for this resistance. Thus, immunoblotting analysis of cell proteins with antibodies against STAT-1 and phosphorylated STAT-1 showed that ESTDAB-004 was not deficient in STAT-1 proteins but that IFN-γ did not induce STAT1 tyrosine phosphorylation (Fig. 2). IFN-γ signaling pathway is initiated upon binding of IFN-γ receptor. IFN-γ binds to extracellular heterodimeric receptor subunits IFN-γ R1 and IFN-γ R2, which are intracellularly associated with Jak-1 and Jak-2, respectively [1,8]. This binding initiates phosphorylation of tyrosine residues in Jak-1, Jak-2, and the cytoplasmic tail of IFN-γ R1. Analysis of the IFN-γ receptors revealed a similar expression in both of the unresponsive cell lines (not shown). Importantly, whereas IFN-α activates Jak-1 and Tyk2, IFN-γ activates Jak-1 and Jak-2. Therefore, we only investigated the phosphorylation of Jak-2, because this kinase is not shared by the IFN-α Jak-STAT pathway. ESTDAB-004 cells expressed Jak-2 protein, but it was only marginally phosphorylated in response to IFN-γ. SOCS1 induction by IFN-γ and negative regulation of IFN-γ signaling have been well documented [20,21]. Moreover, SOCS1 is aberrantly expressed in melanoma cells [22]. Our findings confirm the constitutive expression of SOCS1 protein in ESTDAB-004 cells (Figure 3). We hypothesize that absence of STAT-1 phosphorylation in ESTDAB-004 cells may be mediated via kinase inhibition of Jak-2 by the action of SOCS1 phosphatase.

In contrast, INF-γ treatment increased tyrosine phosphorylation (activation) levels of STAT-1 in ESTDAB-159, the other INF-γ resistant responsive cell line. IRF-1 was also increased, indicating that homodimeric phosphorylated STAT-1 complex is translocated to the nucleus and bound to the GAS sequences in the IRF-1 promoter (Fig 4A). Notably, treatment with IFN-γ produced higher IRF-1 mRNA levels with respect to ESTDAB-056, a melanoma cell line with normal inducibility of MHC class I antigen used as a control. However, none of the IRF-1 associated target genes tested (MHC-class I and iNOS) were induced by IFN-γ in ESTDAB-159 cells.

Pretreatment with all-trans-retinoic acid (atRA) was reported to affect the transcriptional functions of IFN-γ induced IRF-1, inducing its nuclear translocation and enhancing DNA binding activity and transcript levels of IRF-1 target genes [23]. Treatment of ESTDAB-159 cells with atRA did not recover MHC class I inducibility (data not shown). Importantly, point mutation of IRF-1 can abolish IRF-1 DNA binding activity [24]. In acute myeloid leukemia, point mutations or deletions of the IRF-1 gene have frequently been detected [25]. Furthermore, loss of heterozygosity in IRF-1 appears to be critical for the development of human gastric cancers [26,27]. However, this possibility of IRF-1 mutation or transcriptional factor defects was ruled out in the ESTDAB-159 cells. Thus, IFN-γ-induced nuclear translocation and DNA-binding activity of IRF-1 was similar between ESTDAB-159 and control cell line (ESTDAB-056) in an EMSA assay (Fig 5). In addition, sequencing of the complete IRF-1 cDNA revealed no mutation (data not shown).

Available evidence suggests that IRF-1 may function as a tumor-suppressor gene and might induce apoptosis mediated via up-regulation of caspase 8 [28], a key mediator frequently inactivated by epigenetic silencing in many tumors by gene hypermethylation [29,30]. Here, we report that treatment with 5-dAzaC helped to restore IFN-γ-mediated HLA class I inducibility in ESTDAB-159 cells (Fig. 6). We previously reported an epigenetic down-regulation of HLA class I antigens in a melanoma cell line MSR-3, which has hypermethylation of HLA class I genes [31]. However, the MSR-3 cell line did not respond to either IFN α or -γ and exhibited a complete absence of HLA class I expression. The molecular basis of 5-dAzaC activity is different in ESTDAB-159 for several possible reasons. Since ESTDAB-159 cells responded to IFN-α but not to IFN-γ, it is possible that methylation affects the binding of IRF-1 to the ISRE consensus motif in a higher degree than the binding of ISFGF3 induced by IFN-α. Another possibility is that the transactivation of IRF-1 is inhibited by a dominant negative action of several factors [32], which must be regulated by methylation. However these possibilities need to be further investigated.

It summary, IFN-γ exposure in the cancer microenvironment can create conditions for cancer cells to develop alteration in IFN signaling and thereby provide these cells with an adaptive mechanism to survive in hosts with competent immune systems [18]. While IFN-α is used in treatment of cancer with measurable efficacy [33], IFN-γ demonstrated limited success in cancer immunotherapy in humans. It might be explained by tumour-cell insensitivity to IFN-γ [34], or an inability to therapeutically recapitulate the natural periodicity of IFN-γ production. An understanding of the mechanisms by which tumors circumvent cytokine signaling would greatly aid prediction of the immune response in patients treated with IFNs.

## Conclusion

We observed IFN-gamma mediated MHC class I induction in the majority of the melanoma cell lines studied. However, in two cell lines we found different alterations in the IFN-γ signalling pathway. In ESTDAB-004 cells the absence to IFN-γ response was found to be associated with a defect in the earliest steps of the IFN-γ signaling pathway due to absence of STAT-1 tyrosine-phosphorylation. Constitutive SOCS-1 expression may be partially responsible for that. In another cell line, ESTDAB -159, the absence of IFN-γ mediated HLA class I expression might be due to epigenetic blocking of IRF-1 transactivation.

## Competing interests

The author(s) declare that they have no competing interests.

## Authors' contributions

TR carried out the cell culture experiments, flow cytometry analysis of the HLA class I expression and western blots to analyse protein phosphorylation. RM made substantial contributions to conception and design of the study, interpretation of the data. In addition, RM did the mobility shift experiments. ADC carried out culture experiments and the study of HLA class I expression after 5-dAzaC treatments. PJ did the quantitative real-time RT-PCR assays. NA participated in running western blot analysis and contributed to the detailed analysis and interpretation of data. NA also drafted the last versions of the manuscript. FG made substantial contributions to interpretation of data. In addition he critically revised the manuscript. FRC conceived the study and critically reviewed the manuscript. All authors read and approved the final manuscript.

## Pre-publication history

The pre-publication history for this paper can be accessed here:
